# Price increase negotiations to address drug shortages in South Korea’s national health insurance

**DOI:** 10.3389/fphar.2023.1307462

**Published:** 2023-12-04

**Authors:** Seung Rae Yu, Jong Hyuk Lee

**Affiliations:** ^1^ College of Pharmacy, Dong-Duk Women’s University, Seoul, Republic of Korea; ^2^ College of Pharmacy, Chung-Ang University, Seoul, Republic of Korea

**Keywords:** drug shortage, price negotiation, health insurance, budget impact, essential drug

## Abstract

South Korea has adopted a unique approach to address drug shortages by increasing reimbursement prices within its National Health Insurance Service. This study aims to analyze the characteristics, increase rates, affecting factors, and budget impacts of products that have increased price through the negotiation system. Between 2007 and 2022, there were price increase negotiations over 244 items. Of these, price increase negotiations were successful for 217 items, resulting in an agreement rate of 89%. The average rate of price increase for the agreed-upon products was 37.8%, and the overall budget increase for drugs with price increases (*n* = 217) was approximately 24.5%. Budget impact of each variable of the negotiated agreements showed that the number of negotiated agreement items was smaller after 2015 than before 2015, but each total budget impact (initial budget, increased budget, and final budget) and the average budget impact were higher. Although domestic companies accounted for a larger overall budget, the average budget per item was larger for multinational companies. The correlation analysis of the ratio of price increase and budget impact variables showed that the ratio of price increase was positively and significantly correlated with the increased budget, while it was negatively but not significantly correlated with the initial and final budgets. The South Korean model of increasing reimbursement prices in public insurance for drugs at risk of shortages serves as an exemplary case for not only securing patient access but also considering budget management.

## 1 Introduction

Pharmaceuticals are public goods essential for preventing and treating diseases, necessitating a national system for appropriate supply according to demand (Phuong et al., 2019). When supply falls short of demand, resulting in a drug shortage, patients may experience exacerbated conditions and be exposed to side effects due to inappropriate substitute medications (Tucker et al., 2020). Furthermore, societal costs arise from the additional time and effort expended by healthcare providers searching for alternative drugs or treatments, elevating drug shortage from a purely medical problem to a broader societal concern (Pauwels et al., 2014; Postma et al., 2023).

Drug shortages occur globally, irrespective of a nation’s income level (Shukar et al., 2021). Such shortages can be attributed to not only manufacturing and quality issues but also monopolistic market behaviors and difficulties in procuring raw material (Alevizakos et al., 2016; Fox and Tyler, 2017). The globalization of supply chains has also led to transnational propagation of drug shortages (Iyengar et al., 2016). The United States recognized the severity of this issue and enacted the Food and Drug Administration Safety and Innovation Act in 2012 (Chen et al., 2016). This legislation mandates companies to report anticipated drug shortages to the Food and Drug Administration and legally delineates the responsibilities of corporations and the government. Moreover, as countries worldwide experience global crises such as COVID-19, policies to prioritize supplying essential public goods to citizens have been further strengthened, and major pharmaceutical-producing countries have reduced export volumes to prioritize domestic demand (Badreldin and Atallah, 2021). For instance, a global shortage of acetaminophen by the end of 2022 led to competitive procurement efforts among nations. While early reporting of supply disruptions by pharmaceutical companies is crucial, strengthening domestic access to pharmaceuticals through policy support is also essential. A previous study discussed price and reimbursement policies related to drug shortages (Musazzi et al., 2020). Representative examples from Japan and Australia include postponing or exempting drugs with high clinical need from applying the drug price reduction system for a certain period to prevent a drug shortage due to problems with reimbursement prices in public insurance. According to a 2019 survey by the European Association of Hospital Pharmacists, medical professionals have identified price as a significant cause of drug shortages (Miljković et al., 2020). Furthermore, research has shown that if a shortage of specific prescription drugs persists, the risk of drug prices rising along with the disruption of patient care increases (Dave et al., 2018), and the risk of drug shortages increases even when domestic drug prices are significantly lower than those in foreign countries (Alowairdhi et al., 2023).

In this context, raising reimbursement prices within public insurance schemes can be considered a strategy to combat drug shortages. For countries such as South Korea, which operates a single-payer system through the National Health Insurance Service (NHIS), prices and drug shortages are even more closely related. However, no public insurance systems have addressed price increases to alleviate drug shortages. South Korea has been operating a price increase negotiation system for drug shortages since 2007, providing globally relevant insights into tackling drug shortages.

This study aims to analyze the characteristics, rate of price increases, contributing factors, and budget impacts of products that have experienced price hikes through South Korea’s “Price Increase Negotiation System for Drug Shortages.”

When South Korea’s health insurance system was introduced in 2007, the eligibility criteria for price increase negotiations included only clinically essential and irreplaceable drugs. However, owing to growing concerns about drug shortages amid the COVID-19 pandemic, the criteria were expanded in 2020 to include low-cost products for which no alternative with the same active ingredient existed.

**FIGURE 1:**
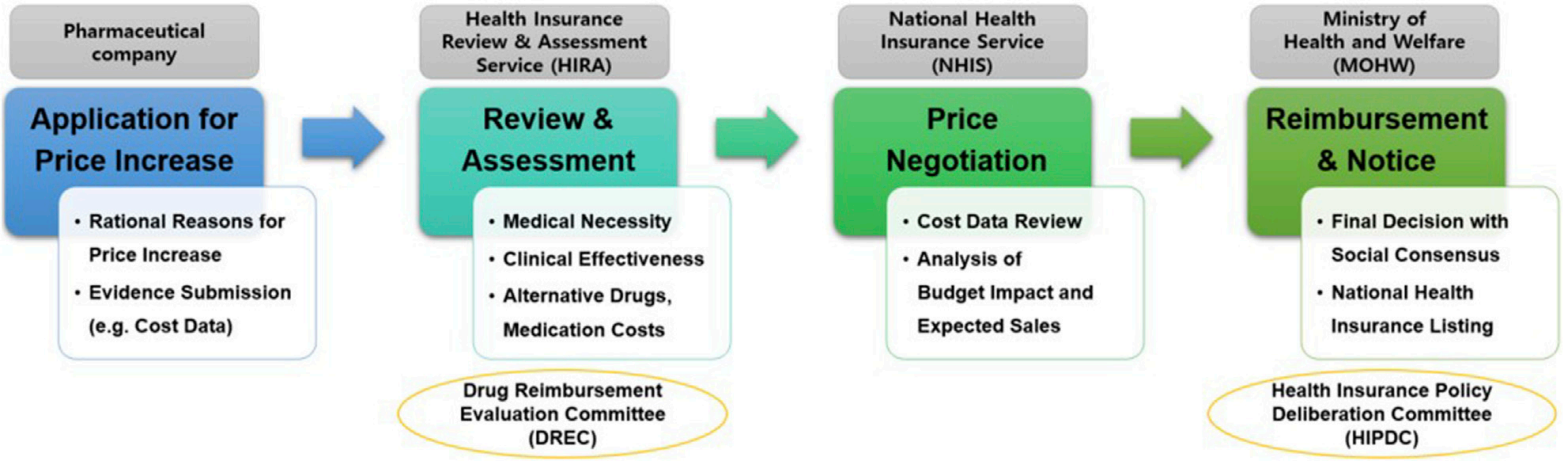
Price increase negotiation process to overcome drug shortages.

Regarding submission documents and procedures for price increase negotiation, pharmaceutical companies must submit a price-increase application along with evidence supporting the need for such an increase, such as production or import costs, to the Ministry of Health and Welfare. Subsequently, the Ministry of Health and Welfare commissions the Health Insurance Review and Assessment Service to review the documents and evaluate the drug’s clinical necessity and utility. If the Health Insurance Review and Assessment Service confirms the need for a price increase, negotiations between the pharmaceutical company and the NHIS determine the final adjusted price (Figure 1).

In other words, South Korea operates a positive list system that selectively covers only drugs that have proven clinical utility and cost-effectiveness. Therefore, even if a drug has a high medical need, the payer does not accept the price increase requested by pharmaceutical companies but adjust the price increase considering the budget impact and supply sustainability. Through this system, South Korea has been able to maintain the cost of pharmaceuticals at a stable level of approximately 24% compared to the total health insurance medical expenses every year (as of 2022, total medical expenses amount to 105.9 trillion won, and pharmaceutical expenses amount to 23.4 trillion won).

## 2 Materials and methods

### 2.1 Data sources

The dataset was constructed by extracting a list of pharmaceutical products that had undergone price increases through the drug price negotiation system introduced in South Korea between January 2007 and December 2022. The data sources included the Health Insurance Drug Benefit List announced by the Ministry of Health and Welfare (HIRA, 2023a), the Drug Payment Adequacy Evaluation Report announced by the Health Insurance Review and Assessment Service (HIRA, 2023b), and the Drug Negotiation Results announced by the NHIS (NHIS, 2023b). Drug-specific indications were verified through the online site, EZDRUG, by the Korea Ministry of Food and Drug Safety (MFDS, 2023). The detailed characteristics of the analyzed drugs subject to analysis were categorized according to the Essential Medicines List provided by the World Health Organization (WHO, 2023b) and the Anatomical Therapeutic Chemical (ATC) Classification System code list (WHO, 2023a). The characteristics of pharmaceutical companies were verified through the websites of various pharmaceutical associations (KPBMA, 2023; KRPIA, 2023), and the financial impact of the price increases was gathered from the HIPDAC review results (MOHW, 2023).

### 2.2 Analysis

We analysed the characteristics of the drugs for which price increases were requested by pharmaceutical companies, accepted by the government, and negotiated. The type of drug supply was categorized into foreign imports and domestic production, so we identified the company type, and the nature of the disease and the degree of patient need were identified by ATC code, such as essential drugs, orphan drugs. We examined variables on the initial price, as the percentage increase in the final price from the initial price is an important measure of budget impact. In consideration of these, we defined the variables as follows:

1. Dependent variable: ratio of price increase = (final price−initial price)/initial price ×100
2. Independent variables: Results: agreement or non-agreement between the pharmaceutical company and the NHIS Structured
- Company type: domestic or multinational
- Essential drugs (EDs): designated as ED or non-ED by the World Health Organization
- Orphan drugs (ODs): designated as OD or non-OD by the Korea Ministry of Food and Drug Safety
- ATC code: World Health Organization’s 1st ATC level (top five codes based on the number of products and the remaining codes)
- Budget: initial budget (expenditure before price increase), final budget (expenditure after price increase), and increased budget (increased expenditure due to price increase). The budget in this study is of the National Health Insurance Service (NHIS).
- Period: 2007–2014 or 2015–2022 (enhancing patient access to drugs for unmet needs began in 2015, including introducing the Managed Entry Agreement system) (Efthymiadou and Kanavos, 2022; NHIS, 2023a)

Descriptive statistics were used for the annual agreement ratio in drug price negotiations, increased price ratio, and budget impact analysis. The Mann-Whitney and Kruskal–Wallis tests were used in independent groups, with comparisons between 2007–2014 and 2015–2022. The analyzed variables were increased price ratios and budget impacts. However, because of the large number of subgroups and small sample sizes in each, an overall test for differences was performed instead of *post hoc* tests for variables with three or more subgroups (e.g., ATC code). Pearson’s correlation analysis was used to examine the relationship between increased price ratios and budget impacts. Multiple linear regression was performed to identify the variables affecting the increased price ratio. The regression analysis was based on the 217 drugs for which NHIS negotiations were agreed upon, and there were no missing values for these 217 drugs. Multiple linear regression analysis was performed and VIF values were calculated for each independent variable to ensure that there were no multicollinearity issues.

Descriptive statistics and statistical significance tests were performed using IBM SPSS version 27.0. All *p*-values were two-tailed, and *p* < 0.05 was considered statistically significant.

## 3 Results

Between 2007 and 2022, there were price increase negotiations over 244 drugs. Of these, price increase negotiations were successful for 217 drugs, resulting in an agreement rate of 89%. The average rate of price increase for the agreed-upon products was 37.8%. During the early years of the system implementation (2009 and 2010), 27 and 53 drugs, respectively, experienced price increases. Notably, in 2022, a significant price surge in 41 drugs was observed, including multiple products containing acetaminophen, affected by COVID-induced supply shortages (Table 1).

**TABLE 1:** The ratios of annual agreement and price increase during price negotiations.

Year	Products with price increase (N)		Agreement ratio (%)	Ratio of price increase			
	Applied	Increased		Mean (%)	SD (%)	Max (%)	Min (%)
2008	8	7	87.5	106.7	0.0	106.7	106.7
2009	32	27	84.4	144.9	24.6	177.0	100.0
2010	59	53	89.8	130.2	22.5	174.0	100.0
2011	15	14	93.3	113.1	8.7	132.8	100.0
2012	17	15	88.2	139.4	18.6	204.6	118.2
2013	13	12	92.3	115.5	8.3	135.8	110.3
2014	6	6	100	124.4	22.1	165.8	106.7
2015	5	5	100	133.0	25.0	176.7	100.0
2016	18	18	100	142.8	44.5	319.9	100.0
2017	2	2	100	305.8	0.0	305.8	305.8
2018	1	1	100	361.5	0.0	361.5	361.5
2019	5	5	100	139.8	36.3	208.9	103.8
2020	9	9	100	182.9	43.3	220.6	100.4
2021	2	2	100	241.7	43.4	285.1	198.2
2022	52	41	78.8	134.3	22.4	176.5	100.0
Total	244	217	88.9	137.8	38.0	361.5	100.0

Note: The values of ratios are expressed in percentage units.

According to the characteristics of the 217 drugs with negotiated price increases, 196 were from domestic companies and 21 were from multinational companies. EDs accounted for 92 drugs, while non-EDs accounted for 125 drugs. Only 19 of these were ODs. According to the ATC code classifications, radioactive diagnostic agents (V) had the highest count of 88, while anti-infectives (J) had the lowest count of 9. Analysis of the rate of price increase according to these variables showed a significant difference after the year 2015 (*p* = 0.001). The rates of price increase were significantly different between ODs and non-ODs (*p* = 0.023). The difference in the rates of price increase for drugs according to the ATC code classification was also statistically significant (*p* = 0.011), and the result of the *post hoc* analysis for each ATC code showed that the difference between blood products (B) and nerve agents (N) was statistically significant (*p* = 0.014; significance values were adjusted by the Bonferroni correction for multiple tests). However, the difference in the rates of price increase for drugs was not statistically significant for two variables: company type (*p* = 0.534) and EDs (*p* = 0.092) (Table 2).

**TABLE 2:** Categorization of variables and ratio of price increase.

Variables		N	Ratio of price increase		*p*-value
			Mean (%)	SD (%)	
Period	Before 2015	134	29.6	23.0	0.001
	After 2015	83	51.1	51.4	
Company type	DM	196	36.3	31.4	0.534
	MN	21	52.4	74.3	
Essential drug	Non-ED	125	37.2	41.7	0.092
	ED	92	38.6	32.4	
Orphan drug	Non-OD	198	39.1	38.6	0.023
	OD	19	24.6	29.1	
ATC code	The rest	21	45.0	57.4	0.011
	J	9	27.9	10.2	
	N	19	48.0	20.4	
	A	24	26.7	21.6	
	B	56	27.5	21.8	
	V	88	44.5	45.4	
Total		217	37.8	38.0	

DM, domestic company; MN, multinational company; ED, essential drug; OD, orphan drug.

ATC, code: A, alimentary tract and metabolism; B, blood and blood forming organs.

J, antiinfectives for systemic use; N, nervous system; V, various.

The analysis results of the budget impact of each variable of the negotiated agreements showed that the number of negotiated agreement items was smaller after 2015 than before 2015, but both the total budget amount (initial budget, increased budget, and final budget) and the average budget impact of for each item were higher.

And the average budget impact was significantly different (*p* = 0.002, 0.001, and 0.001, respectively). Although domestic companies accounted for a larger overall budget, the average budget per item was larger for multinational companies (*p* = 0.001, 0.087, and 0.001). Additionally, non-EDs had larger overall and per-item budgets, except in the case of increased budgets, in which EDs were higher (*p* = 0.025, 0.003, and 0.028). Although not statistically significant (*p* = 0.130, 0.059, and 0.184), non-ODs had larger initial budgets and increased budgets than ODs, and ODs had larger final budgets than non-ODs. Overall, the differences in the ATC codes were significant (*p* < 0.001, 0.001, and 0.004). The total financial amount was largest for radioactive diagnostic agents (V), and the average financial size per item was largest for anti-infectives (J). The result of the *post hoc* analysis of each ATC code showed that the difference between radioactive diagnostic agents (V) and digestive metabolites (A) was significant (*p* = 0.006, 0.018) in the case of the initial budget, and the differences between radioactive diagnostic agents (V) and digestive metabolites (A) and between radioactive diagnostic agents (V) and anti-infectives (J) were significant (*p* = 0.018, 0.041) in the case of the final budget. However, no ATC code combination with a significant difference was identified in the case of increased budget (Table 3).

**TABLE 3:** Budget impact according to the variables.

Variables		N	Initial budget			p	Increased budget			p	Final budget			p
			Sum	Mean	SD		Sum	Mean	SD		Sum	Mean	SD	
Period	Before 2015	134	187,199	1,248	4,214	0.002	42,826	320	615	0.001	230,025	1,717	4,839	0.001
	After 2015	83	222,586	2,368	5,750		57,489	693	1,391		280,075	3,374	7,176	
Company type	DM	196	347,195	1,607	4,991	0.001	86,421	441	1,001	0.087	433,616	2,212	5,977	0.001
	MN	21	62,590	2,235	4,028		13,894	662	1,002		76,484	3,642	4,941	
Essential drug	Non-ED	125	256,319	1,792	4,992	0.025	54,496	436	846	0.003	310,815	2,487	5,778	0.028
							45,820	498	1,184		199,285	2,166	6,056	
	ED	92	153,465	1,519	4,747									
Orphan drug	Non-OD	198	363,959	1,701	5,061	0.130	95,867	484	1,040	0.059	459,826	2,322	6,041	0.184
							4,448	234	422		50,274	2,646	4,143	
	OD	19	45,826	1,528	3,475									
ATC code	The rest	21	92,196	3,842	7,257	<0.001	10,120	482	763	0.001	102,316	4,872	8,142	0.004
							14,361	1,596	3,055		74,625	8,292	16,879	
	J	9	60,264	5,479	12,772									
	N	19	21,464	1,073	1,322		13,670	719	995		35,134	1,849	2,322	
	A	24	44,384	1,775	2,492		10,343	431	473		54,727	2,280	2,902	
	B	56	86,813	1,336	5,210		11,297	202	476		98,110	1,752	5,959	
	V	88	104,663	1,057	2,262		40,524	461	884		145,187	1,650	2,985	
Total		217	409,785	1,679	4,894		100,315	462	1,003		510,100	2,351	5,900	

Note: The value of the budget variable was calculated in million KRW (US$ 1,200 = 1 mil. KRW).

The correlation analysis of the ratio of price increase and budget impact variables showed that the ratio of price increase was positively and significantly correlated with the increased budget (*p* = 0.005), while it was negatively but not significantly correlated with the initial and final budgets (Table 4). Although not statistically significant, drugs at risk of running out tended to have a lower ratio of price increase when their initial budget was higher.

**TABLE 4:** Correlation analysis of ratio of price increase and budget impact.

Variables	Increased price ratio	Initial budget	Increased budget	Final budget
Ratio of price increase	1			
Initial budget	−0.129	1		
Increased budget	.196**	.703***	1	
Final budget	−0.079	.993***	.784**	1

*p < .05, **p < 0.005, ***p < 0.001.

Using multiple linear regression with the ratio of price increase as the dependent variable, the full model indicated that the period (pre- and post-2015), company type, and initial budget were significant predictors. To assess the goodness of fit of the regression model, we conducted an ANOVA analysis to test the significance of the F-value and presented the explanatory power of each model using the adjusted *R* ^2^ value. The model’s F(p) value was 3.871, and the adjusted *R* ^2^ was 0.117. The variables of period, initial budget, and company type were sequentially selected in the stepwise model, and ATC V code was added to Model 4. The F(p) value was 17.644, and the adjusted *R* ^2^ was 0.072 (Table 5). As a result, the rate of price increase for drugs at risk of shortage was higher after 2015 (post-2015) and for multinational companies and drugs with ATC V code indications than for domestic companies and other ATC code indications. However, a higher initial budget was correlated with a lower rate of price increase.

**TABLE 5:** Multiple linear regression analysis of the ratio of price increase.

Variables	Full model			Stepwise model							
				Model 1		Model 2		Model 3		Model 4	
	*β*	t	VIF	*β*	t	*β*	t	*β*	t	*β*	t
(Constant)		12.553 ***			40.820 ***		40.934 ***		39.658 ***		32.022 ***
Period = After 2015	0.245	3.287 **	1.362	0.275	4.200 ***	0.295	4.527 ***	0.297	4.585 ***	0.286	4.438 ***
Company = MN	0.177	2.621 **	1.111					0.138	2.143 *	0.167	2.557 *
ED = ED	0.125	1.412	1.913								
OD = OD	−0.027	−0.344	1.561								
ATC = J	−0.055	−0.709	1.453								
ATC = N	−0.027	−0.300	1.978								
ATC = A	−0.089	−0.950	2.159								
ATC = B	−0.105	−0.902	3.318								
ATC = V	0.122	0.956	3.981							0.144	2.189 *
Initial budget	−0.146	−2.180*	1.104			−0.164	−2.520 *	−0.174	−2.685 *	−0.159	−2.454 *
F(p)	3.871***			17.644***		12.218***		9.812***		8.688***	
Adj. R2	0.117			0.072		0.094		0.109		0.125	
Durbin-Watson	1.016			0.969							

*p < .05, **p < 0.005, ***p < 0.001.

Note: Dependent variable: ratio of price increase.

Reference group: Period*before 2015, Company*DM, ED*non-ED, OD*non-OD, ATC*the rest.

## 4 Discussion

This study examined the South Korean case in which the government increased drug reimbursement prices in public insurance as an institutional solution to pharmaceutical shortages. This study identified which drugs were subject to this regulatory change and the resulting rates of price increase negotiated between pharmaceutical companies and payers. Budget impacts and factors affecting these price increases were also evaluated.

From 2007 to 2022, the average rate of agreement in price increase negotiations for drugs was 89%. This high rate indicates a consensus among stakeholders regarding the need and utility of a price negotiation mechanism to resolve drug shortages. In South Korea, there are 25,000 drugs that are covered by health insurance as of 2022, but the number of drugs for which price increase applications have been accepted and negotiated (*n* = 244) is very small. This is because the government agency strictly evaluates the appropriateness of the price increase as well as the need for supply, and most of the drugs that passed HIRA’s evaluation (*n* = 244) were agreed upon in negotiations with NHIS (*n* = 217). Some failed negotiations (*n* = 27) were primarily due to the policy’s early years (2008–2013), and recently, in 2022, price negotiations were conducted for several mild disease (e.g., anti-inflammatory) drugs that are expected to be out of stock due to the impact of COVID-19.

Domestic companies had a higher number of items subject to price increase than multinational ones. This is likely due to the higher number of domestic companies than multinational ones. South Korea’s pharmaceutical market is one of the largest in Asian countries after China and Japan, but it still relies heavily on multinational pharmaceutical companies for innovative new drugs or treatments for serious diseases. As the business model of domestic pharmaceutical companies is mainly in the generics sector, there are many cases where the original developer of the drug or the supply route in South Korea is a multinational pharmaceutical company, which raises patient access issues due to supply shortages. In order to stabilize the supply of drugs, it is necessary to improve the production infrastructure of essential drugs by domestic pharmaceutical companies in the medium to long term.

When categorized by disease characteristics, non-EDs and non-ODs were more prevalent. While previous studies have emphasized the importance of drug shortages in acute, severe, and rare diseases (Jarosławski et al., 2017), the findings of this study confirm that South Korea is addressing shortages for less severe and rare diseases through public measures. Furthermore, the application of the system and rate of price increase differed according to the therapeutic class (ATC code). According to previous studies, there is a shortage of anti-infectives (J) in the United States, and in Europe, anti-infectives (J) and nerve agents (N) are highly out of stock (Griffith et al., 2012; Turbucz et al., 2022). In other words, the pattern of drug shortage may differ depending on the country. This study showed that the risk of shortage due to the low prices of radioactive diagnostic agents (V) and blood products (B) was highest in South Korea. Previous research in Canada suggested that shortage management is necessary for drugs in the ATC classification system, including sensory drugs, which have a high patient demand and risk of being out of stock (Zhang et al., 2020). These findings imply that each country should assess its situation and apply realistic solutions, such as price increases. While our literature review identified out-of-stocks for conditions with relatively high prevalence in the U.S., Canada, and Europe, such as neurological and respiratory, we also identified out-of-stocks for specialty ATC codes such as radiopharmaceuticals and blood products. These differences may be due to the fact that price increases are driven by the availability of clinically substitutable drugs in the same class or family of drugs, not simply by a shortage of a particular drug.

From the payer’s perspective, increasing drug prices to address shortages results in additional financial burden. Analysis of the budget impacts indicates that various conditions affect price negotiation outcomes. Both the total expenditure scale and expected prices have increased since 2015, implying that shortages of high-expenditure drugs are becoming more prevalent. Although the total expenditure was higher for domestic companies, the average scale per drug was higher for multinational companies. This finding suggests that if the price of drugs from multinational companies increases more frequently, the total expenditure scale could increase more quickly. Although the total expenditure scale may be smaller, the average scale may be similar or even higher for EDs and ODs listed by the World Health Organization, thus, requiring careful consideration of the fiscal impact and patient safety when resolving drug shortages. This study confirmed that drugs for diseases in the ATC classification system had a significant impact on the total budget impact and the average value per drug, suggesting the need to proactively monitor and respond to price increases to resolve drug shortages in diseases with a relatively large budget impact. The factor that ultimately showed the most significant causal relationship among the various independent variables included in the multiple regression analysis was summarized as the introduction of the government’s coverage-enhancing policy variable (period), the type of pharmaceutical supplier (company type), and the distribution of therapeutic agents for specific conditions (ATC code). Regression analysis confirmed that the rate of price increase was positively correlated with the price increase negotiations for drugs by multinational companies after 2015 and was particularly high for radioactive diagnostic agents (V) compared to other ATC codes. Conversely, this study found a negative relationship between the initial expenditure scale and rate of price increase, indicating that South Korea’s price increase negotiation system for drugs is trying to prevent stockouts and reduce the impact on insurance finances.

Unlike administrative dispositions based on laws and regulations, negotiations involve a variety of decision-making factors, including the purchasing power of the parties, which are difficult to quantify with objective indicators. In the case of drug price negotiations, additional explanatory variables may include the foreign listing status of the drug, the listed price, and the cost level (profit margin) that caused the shortage in Korea. However, since these variables cannot be accurately identified except by the pharmaceutical company that owns the product or the negotiating party, it is necessary for government agencies to conduct further public research from the perspective of system improvement.

It should be noted that drug shortages are also related to supply environment and economic factors. In terms of supply environment, shortages are more likely to occur in markets with a single generic manufacturer, so health authorities need to regularly monitor ingredients that are lacking in late entrants and motivate pharmaceutical companies to participate in generic development (Zhang et al., 2020). In addition, economic factors such as low margins, small market size, and rising costs of raw materials are also factors that lead to drug shortages (Shukar S et al., 2021). In particular, for drugs with low sales revenue, there is a threshold where pharmaceutical companies cannot be forced to supply, so the Korean price increase system discussed in this study has the potential to be a realistic alternative to resolve drug shortages. Despite these findings and interpretations, this study has several limitations. A comparative analysis of countries operating similar or identical systems can provide more generalizable findings. However, this study only deals with the South Korean system and its outcomes, necessitating further research with international comparisons. Furthermore, the regression model employed in this study, which uses the rate of price increase as the dependent variable, has limited explanatory power. This limitation likely arises owing to various factors affecting price negotiations between pharmaceutical companies and governments. Future research should incorporate more factors and cases to improve the explanatory power of this model. Finally, this study used estimated drug costs at the price increase negotiation phase as the final budget, which may differ from actual consumption and, thus, may have some error. Future research should provide more insights into patient accessibility and financial sustainability if the drug price increase negotiation system can further confirm the extent to which the actual drug costs match the initially expected drug costs.

As countries have different healthcare systems for drugs prone to shortages, it is essential to operate a suitable system in a timely manner. International cooperation is crucial in addressing global drug shortages. The South Korean model of increasing reimbursement prices in public insurance for drugs at risk of shortages serves as an exemplary case for not only securing patient access but also considering budget management. Sharing these results can provide critical insights into proactive responses to international drug shortages. Ongoing research is warranted to explore various options for addressing drug shortages worldwide.

## Data Availability

The raw data supporting the conclusion of this article will be made available by the authors, without undue reservation.
